# A homozygous stop-gain variant in *ARHGAP42* is associated with childhood interstitial lung disease, systemic hypertension, and immunological findings

**DOI:** 10.1371/journal.pgen.1009639

**Published:** 2021-07-07

**Authors:** Qifei Li, Michal Dibus, Alicia Casey, Christina S. K. Yee, Sara O. Vargas, Shiyu Luo, Samantha M. Rosen, Jill A. Madden, Casie A. Genetti, Jan Brabek, Catherine A. Brownstein, Shideh Kazerounian, Benjamin A. Raby, Klaus Schmitz-Abe, John C. Kennedy, Martha P. Fishman, Mary P. Mullen, Joan M. Taylor, Daniel Rosel, Pankaj B. Agrawal

**Affiliations:** 1 Division of Newborn Medicine, Boston Children’s Hospital and Harvard Medical School, Boston, Massachusetts, United States of America; 2 Division of Genetics and Genomics, Boston Children’s Hospital and Harvard Medical School, Boston, Massachusetts, United States of America; 3 The Manton Center for Orphan Disease Research, Boston Children’s Hospital and Harvard Medical School, Boston, Massachusetts, United States of America; 4 Department of Cell Biology, Charles University in Prague, Viničná 7, Prague, Czech Republic; 5 Department of Cell Biology, Biotechnology and Biomedicine Centre of the Academy of Sciences and Charles University (BIOCEV), Průmyslová 595, Vestec u Prahy, Czech Republic; 6 Division of Pulmonary Medicine, Boston Children’s Hospital and Harvard Medical School, Boston, Massachusetts, United States of America; 7 Division of Immunology, Boston Children’s Hospital and Harvard Medical School, Boston, Massachusetts, United States of America; 8 Department of Pathology, Boston Children’s Hospital and Harvard Medical School, Boston, Massachusetts, United States of America; 9 Channing Division of Network Medicine, Department of Medicine, Brigham and Women’s Hospital and Harvard Medical School, Boston, Massachusetts, United States of America; 10 Pulmonary and Critical Care Medicine, Department of Medicine, Brigham and Women’s Hospital and Harvard Medical School, Boston, Massachusetts, United States of America; 11 Department of Cardiology, Boston Children’s Hospital and Harvard Medical School, Boston, Massachusetts, United States of America; 12 Dept. Pathology and Laboratory Medicine, University of North Carolina, Chapel Hill, North Carolina, United States of America; University of Alabama at Birmingham, UNITED STATES

## Abstract

*ARHGAP42* encodes Rho GTPase activating protein 42 that belongs to a member of the GTPase Regulator Associated with Focal Adhesion Kinase (GRAF) family. ARHGAP42 is involved in blood pressure control by regulating vascular tone. Despite these findings, disorders of human variants in the coding part of *ARHGAP42* have not been reported. Here, we describe an 8-year-old girl with childhood interstitial lung disease (chILD), systemic hypertension, and immunological findings who carries a homozygous stop-gain variant (c.469G>T, p.(Glu157Ter)) in the *ARHGAP42* gene. The family history is notable for both parents with hypertension. Histopathological examination of the proband lung biopsy showed increased mural smooth muscle in small airways and alveolar septa, and concentric medial hypertrophy in pulmonary arteries. *ARHGAP42* stop-gain variant in the proband leads to exon 5 skipping, and reduced ARHGAP42 levels, which was associated with enhanced RhoA and Cdc42 expression. This is the first report linking a homozygous stop-gain variant in *ARHGAP42* with a chILD disorder, systemic hypertension, and immunological findings in human patient. Evidence of smooth muscle hypertrophy on lung biopsy and an increase in RhoA/ROCK signaling in patient cells suggests the potential mechanistic link between ARHGAP42 deficiency and the development of chILD disorder.

## Author summary

Childhood interstitial lung disease (chILD) is a heterogeneous group of rare disorders characterized by diffuse pulmonary infiltrates, respiratory signs and symptoms, and impaired gas exchange. These disorders are complex to diagnose and are associated with substantial morbidity and mortality. Although pathogenic variants in a number of genes have been described to cause chILD, these known genetic etiologies explain only a minority of the cases and there are additional genes yet to be identified. We have identified an 8-year-old girl with chILD, systemic hypertension, and immune abnormalities who carries a homozygous stop-gain variant in the *ARHGAP42* gene. Functional studies demonstrate that this stop-gain variant leads to exon 5 skipping and reduced levels of ARHGAP42 protein. We also show enhanced RhoA expression and its activity in the patient’s lymphoblastoid cell lines. ARHGAP42 is involved in regulation of blood pressure and its deficiency causes hypertension in murine models and human adults. This is the first report to link a homozygous stop-gain variant in *ARHGAP42* with a chILD disorder, systemic hypertension, and immunological findings in a pediatric patient. Identification of additional chILD patients carrying *ARHGAP42* mutations will better define its role in chILD.

## Introduction

ARHGAP42 (Rho GTPase Activating Protein 42), also known as GRAF3, is a member of the GRAF (GTPase-activating protein for Rho associated with focal adhesion kinase) family of Rho-specific GAP (GTPase-activating protein). It is highly expressed in the smooth muscle cell (SMC) layers of blood vessels, stomach, intestine, and lung [1]. Previous reports have shown that several single nucleotide polymorphisms (SNPs: rs604723, rs633185, rs607562, and rs667575) of *ARHGAP42* are blood pressure (BP)-associated loci [1–4]. *Arhgap42*-deficient mice, homozygous for a gene-trap-mediated reduction in *Arhgap42* mRNA levels, exhibit significant hypertension with no other manifestations [1]. The *Arhgap42* heterozygous mice also display significant hypertension [1,5]. RhoA kinase inhibitor can abrogate this response in these *Arhgap42*-deficient mice [1,5]. Functional studies indicated that ARHGAP42 acts preferentially as a GAP for RhoA and that it plays a key role in maintaining normal BP homeostasis by reducing RhoA-dependent phosphorylation of the myosin light chain (MLC) and Ca2^+^-mediated SMC contractility in resistance vessels [1,6]. Recently, a SNP (rs633185) of *ARHGAP42* has been reported to be associated with chronic obstructive pulmonary disease [7], although the relationship between *ARHGAP42* and lung diseases has not yet been elucidated.

Although much effort has been made to understand the mechanism of ARHGAP42 in hypertension, its effect on childhood interstitial lung disease (chILD) and immune abnormalities has not been described. Here, we report an 8-year-old girl with chILD and persistent lymphocytosis who carries a homozygous stop-gain variant in *ARHGAP42* (NM_152432.2:c.469G>T, p.(Glu157Ter)). To determine the pathogenicity of this variant, we performed anatomic and functional studies to link it with the patient’s phenotype.

## Results

### Clinical history

The proband, a female child, with a birth weight of 2,780 g (16th percentile), was born at 39 weeks gestation following an uncomplicated pregnancy, via vaginal delivery through meconium.

### Childhood interstitial lung disease (chILD)

**Fig 1:**
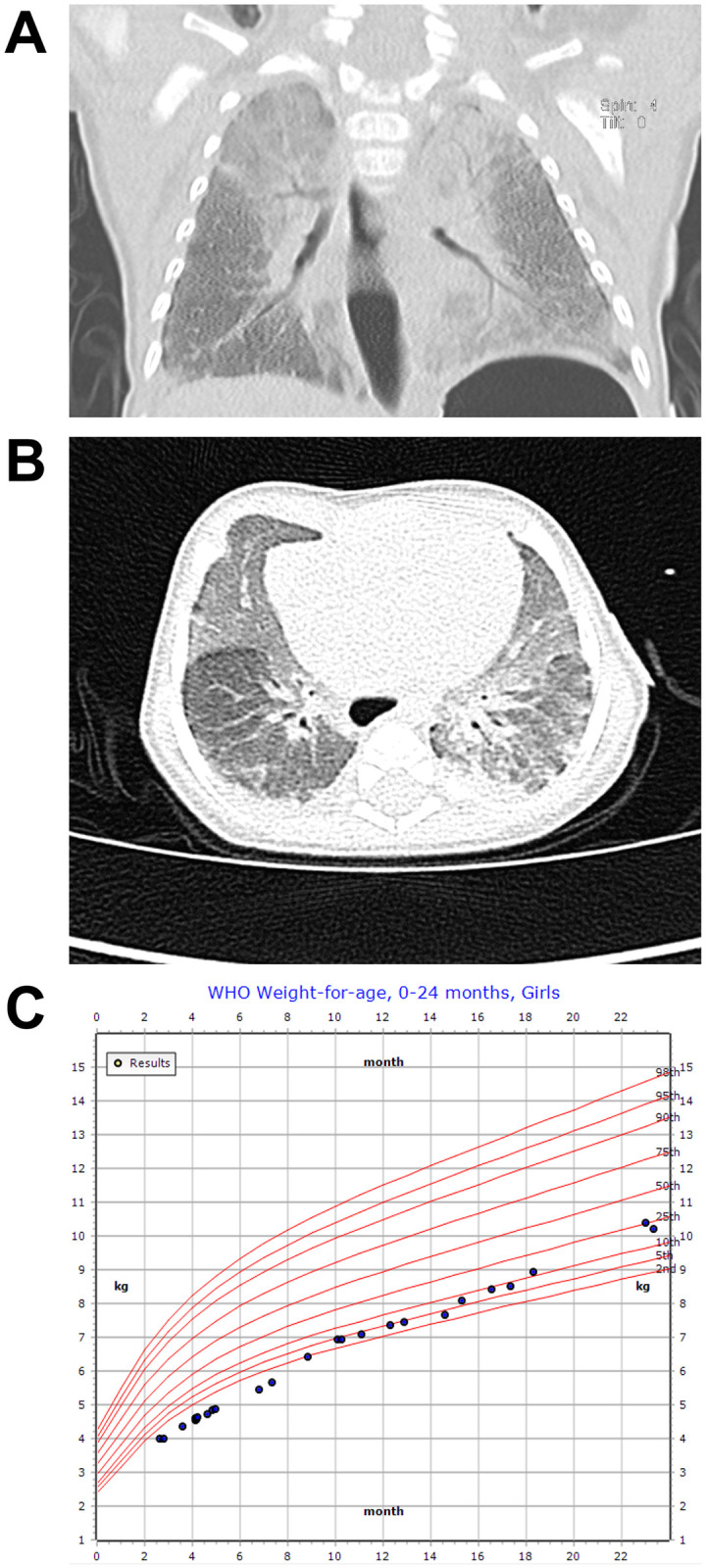
Clinical findings of the proband with homozygous *ARHGAP42* stop-gain variant. (A) Chest X-ray at age 3 months showed bilateral diffuse opacities and low lung volumes; (B) Initial chest CT scan at age 3 months displayed diffuse ground glass opacities with subpleural sparing and patchy subpleural opacities; (C) Patient growth chart for weight through two years of age.

The infant had tachypnea with normal oxygen saturation soon after birth and was discharged home. Poor feeding, failure to thrive, and labored breathing developed soon after. Evaluation at 2 months of age showed retractions, tachypnea, and rales needing hospitalization. Oxygen saturations were in the low 80’s on room air and required 0.5 L/min supplemental oxygen by nasal cannula to normalize. A chest X-ray (Fig 1A) demonstrated bilateral diffuse opacities and low lung volumes, concerning for ILD. A chest computed tomography (CT) scan (Fig 1B) at age 3 months showed diffuse ground glass opacities with subpleural sparing and patchy subpleural opacities in the dependent portions of both lower lobes; air trapping was not observed. Serial echocardiograms revealed no evidence of pulmonary hypertension or structural heart disease. The patient was treated with oxygen and required bi-level positive airway pressure (BiPAP) at night to maintain normocarbia. She also had aspiration during swallow and was treated with thickened feeds.

The child subsequently demonstrated a gradual, yet sustained, improvement and near-complete resolution of her lung disease over the subsequent 8 years of follow-up. She had no history of recurrent pneumonia or frequent lower respiratory tract infections. Her weight recovered to the 10^th^ percentile by age 18 months with nutritional supplementation (Fig 1C). She was weaned off of daytime oxygen at two years of age, and she continued nighttime oxygen therapy until she was 4 years and 9 months old. Repeat chest CT showed radiographic improvement in her lung disease. At her most recent follow-up at age 8 years, she showed continued clinical stability, with normal body mass index, excellent interval linear growth, regular participation in multiple athletic interests, and rare use of nighttime supplemental oxygen with only occasional upper respiratory tract infections.

### Pathological findings

**Fig 2:**
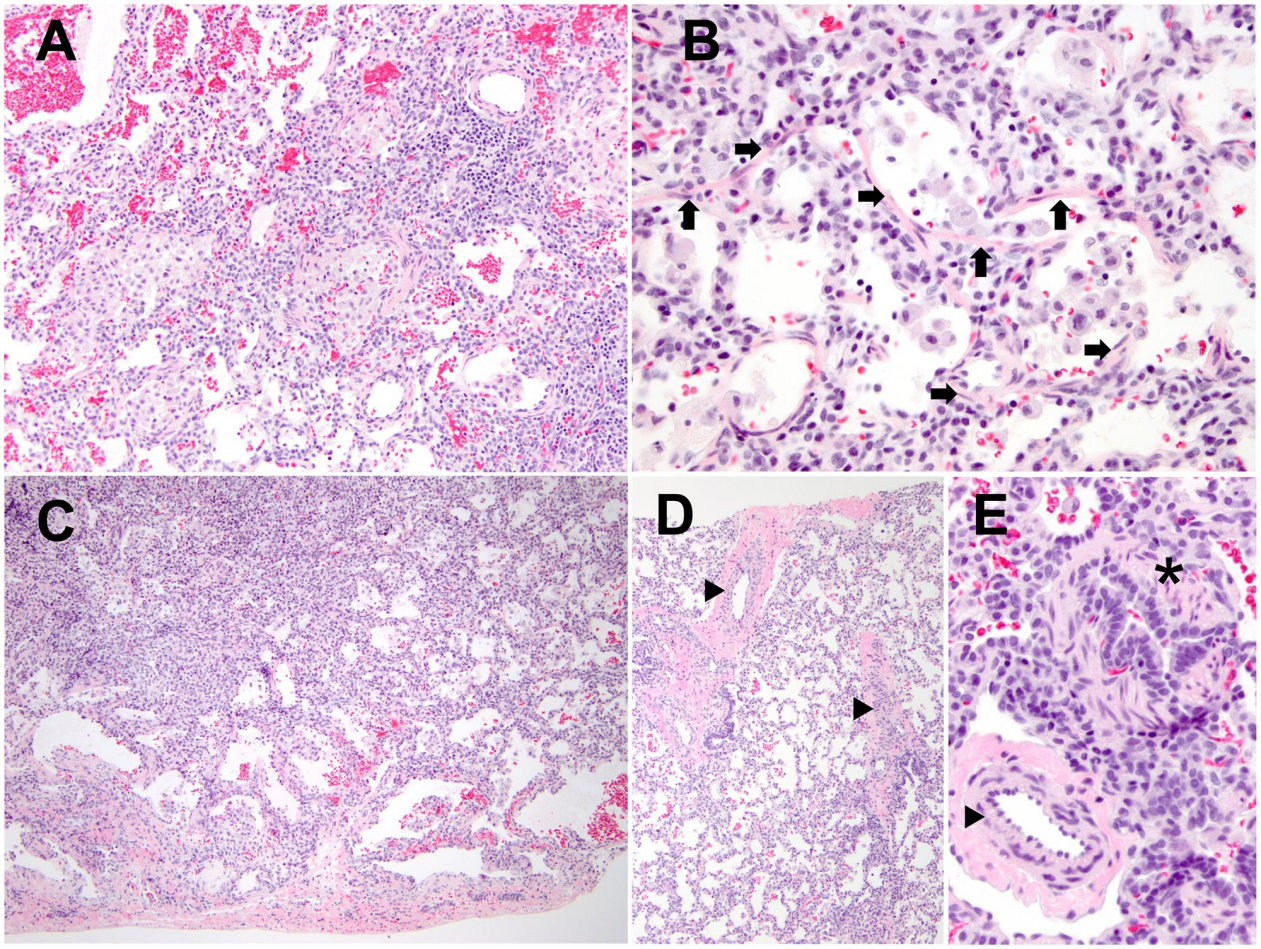
Pathological findings of the proband with homozygous *ARHGAP42* stop-gain variant. Lung histologic findings at age 4 months, by hematoxylin and eosin stain: (A) Small airway obstructive-type changes, with prominent mural smooth muscle and intraluminal macrophages (original magnification, 200x); (B) Intra-alveolar septal extension of smooth muscle (arrows) (original magnification, 400x); (C) Large/maldeveloped airspaces, accentuated subpleurally (original magnification, 100x); (D) Concentric medial hypertrophy in pulmonary arteries (arrowheads) (original magnification, 100x); (E) Pulmonary artery (arrowheads) and airway (asterisk), both with prominent smooth muscle (original magnification, 400x).

Left lung wedge biopsies, obtained from 4 different sites at age 4 months, demonstrated abnormalities in multiple compartments of the lung (Fig 2). Small airways showed increased mural smooth muscle; intraluminal macrophages were often present, and bronchus-associated lymphoid tissue was prominent (Fig 2A). Alveolar septa were widened by extension of airway smooth muscle as well as by patchy pulmonary interstitial glycogenosis and a minor component of type II pneumocyte hyperplasia (Fig 2B). There was increased variation in alveolar size, with enlargement most prominent subpleurally (Fig 2C). A subset of pulmonary arteries showed mild concentric smooth muscle hypertrophy (Fig 2D and 2E).

### Immunological findings

The proband was noted to have persistent leukocytosis and lymphocytosis since the age of 3 months (S1 Fig). Absolute lymphocyte count (ALC) and total white blood cells (WBC) were persistently high or above normal for age, with normal numbers of circulating neutrophils, monocytes, and eosinophils. By 5 years of age, the proband had developed extensive verrucae vulgares on her hands and lip. Limited immunologic phenotyping at age 7 showed that the patient’s lymphocytosis was comprised of proportionately elevated numbers of T cells, B cells and NK cells, which were all present at ~2x the upper normal range of absolute cell counts for age. The CD4^+^ T cell population showed a slightly high percentage of recent thymic emigrant (RTE) cells (66.3% CD4^+^ CD31^+^ CD45RA^+^, normal range: 45.3%-63.6%), but this did not correlate with an increase in the proportion of naïve CD4^+^ T cells. Percentages of naïve vs. memory/effector CD4^+^ and CD8^+^ T cells were normal, except for a slightly low proportion of TEMRA CD8^+^ T cells (8.7% CD45RA^+^ CCR7^+^, normal range: 9.1%-49.1%). Functional T cell testing showed normal lymphocyte proliferation to stimulation with mitogens (PHA, ConA and anti-CD3) and antigens (Candida and Tetanus). Neutrophil oxidative function (DHR) was normal.

Analysis of circulating B cell populations showed slightly high proportions of naïve B cells (78.1% CD19^+^CD27^-^IgD^+^, normal range: 47.3%-77%), with slightly low proportions of transitional B cells (6.7% CD24^hi^ CD38^hi^, normal range: 7.2%-23.8%) and plasmablasts (0.1% CD24^low^ CD38^high^, normal range: 0.4%-5.2%). IgG, IgE and IgA levels were normal for age at 5 years. Although she had received all recommended vaccinations, at 7 years, she had non-protective specific IgG antibody responses for hepatitis B surface antibody and 9/12 Streptococcus pneumoniae serotypes. She did have a protective tetanus IgG titer at the same time point. Vaccine challenges were not performed.

### Systemic hypertension

The proband was noted to have intermittent systemic hypertension. An ambulatory blood pressure monitoring was performed at 8 years of age, which showed both mean 24-hour systolic (119 mmHg, 95%ile) and diastolic (75 mmHg, 95%ile) ambulatory blood pressure to be at or above the 95%ile for sex-age (S1 Table).

The family history is notable for father developing hypertension in his 20s, and the mother with a recent diagnosis of hypertension. Both are under no treatment so far although they are being monitored closely. Both parents have a history of exercise-induced asthma during childhood. In addition, the proband’s father had recurrent streptococcal pharyngitis during childhood, multiple antibiotic reactions and gastroesophageal reflux. The mother is recently diagnosed with autoimmune sclerosis of her left eye and treated with adalimumab (Humira). The paternal grandfather has a history of recurrent bronchitis and pneumonia and asthma since childhood, as well as uncontrollable hypertension beginning in his mid-50s. He is currently on losartan and amlodipine. The maternal grandfather also has hypertension and is on medication, but no further information is available.

### A homozygous stop-gain variant in *ARHGAP42* identified by exome sequencing (ES)

**Fig 3:**
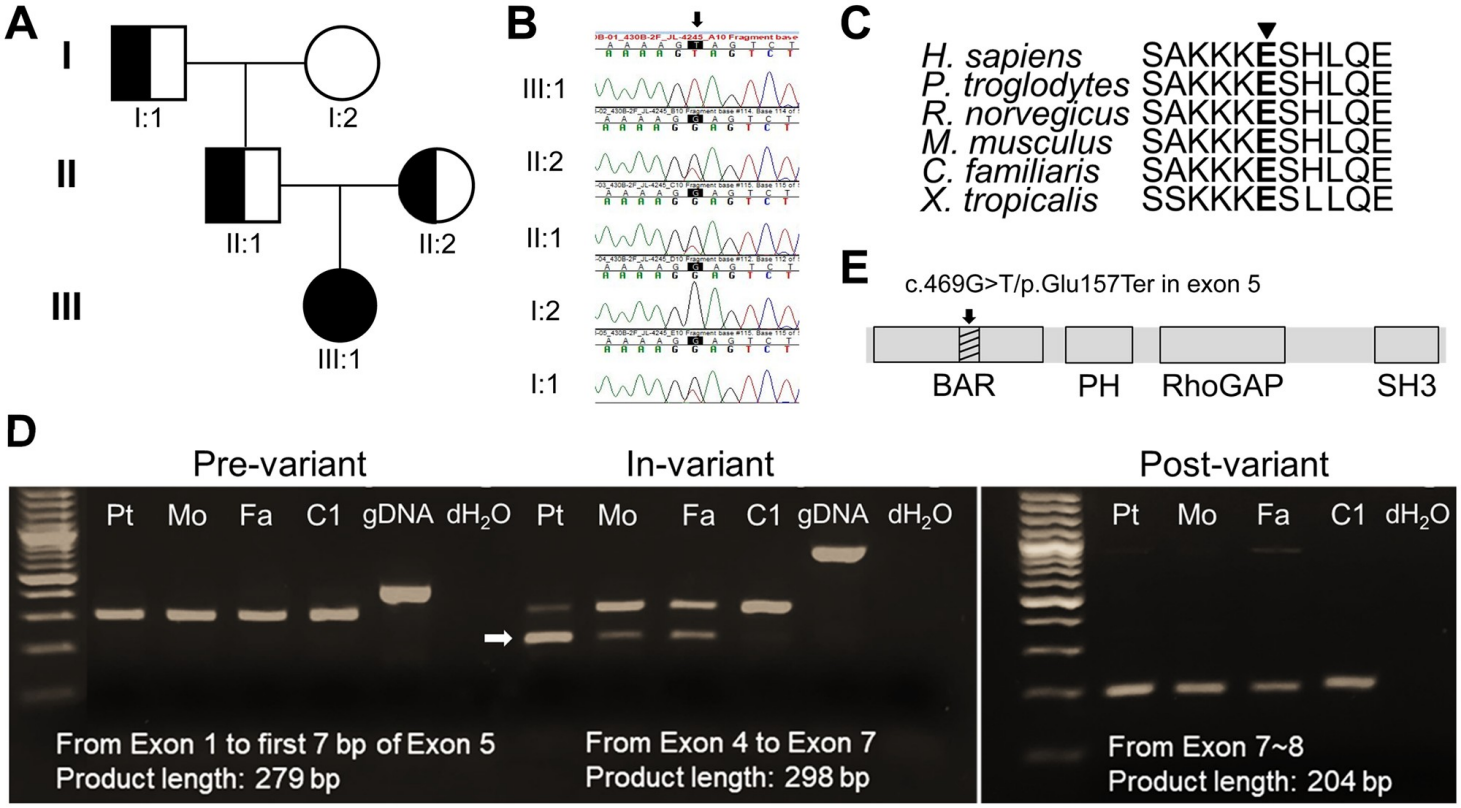
Genetic findings in the family with *ARHGAP42* stop-gain variant. (A) Pedigree of the family carrying *ARHGAP42* variant. Half-filled symbol: heterozygous; Filled symbol: homozygous; (B) Sanger sequencing chromatogram for the family of the *ARHGAP42* variant; (C) Amino acid 157 (arrowhead) is evolutionally conserved in vertebrates; (D) Agarose gel electrophoresis result of *ARHGAP42* pre-variant, in-variant, and post-variant amplification by PCR in cDNA samples extracted from blood. The smaller band (arrow) corresponds with exon 5 deletion confirmed by Sanger sequencing. Pt: patient; Mo: mother; Fa: father; C1: control; gDNA: genomic DNA. Pre-variant: Exon 1 to first 7 bp of Exon 5, 279 bp for *ARHGAP42* cDNA. A 373 bp band seen with gDNA is from non-specific amplification of another locus; In-variant: Exon 4 to Exon 7, 298 bp for *ARHGAP42* cDNA. A 618 bp band was seen with gDNA from non-specific amplification from another locus; Post-variant: Exon 7~8, 204 bp for *ARHGAP42* cDNA. (E) Schematic of ARHGAP42 functional domains. The amino acid change is depicted by an arrow, and the skipped exon 5 is depicted as a shaded box in the BAR domain.

The pedigree of the family is shown in Fig 3A. Clinical testing for common genetic causes of chILD, including genes encoding the ATP-binding cassette transporter A3 (ABCA3) and the surfactant proteins (SFTPB and SFTPC), was negative. Trio ES was performed and a novel homozygous variant in *ARHGAP42* (hg19, chr11:100784267, NM_152432.2:c.469G>T, p.(Glu157Ter)) was identified in the proband (III:1) and confirmed by Sanger sequencing (Fig 3B). While the family is not known to be consanguineous, the parents shared 0.7% of the genome of which the 19.72 MB region containing *ARHGAP42* was the largest (hg19, Chr11: 82924358–102649954). There are two other smaller shared regions of 4.8 Mb and 3.2 Mb on chromosomes 2 and 15 respectively. Additional homozygous variants were evaluated and no additional candidate variant was identified. Both parents (II:1 and II:2) and the paternal grandfather (I:1) were heterozygous for this variant.

This variant is absent from both the publicly available Exome Aggregation Consortium (ExAC) and genome aggregation database (gnomAD) databases, and is classified as damaging by multiple *in silico* prediction software packages, including MutationTaster and Combined Annotation-Dependent Depletion (CADD). The probability of being Loss-Of-Function (LOF) Intolerant (pLI) score for *ARHGAP42* in gnomAD is 1, indicating that the *ARHGAP42* cannot tolerate protein truncating variation. In addition, no homozygous LOF variant is present in the ExAC or gnomAD databases. The glutamate 157 residue is highly conserved in vertebrates (Fig 3C). No additional *de novo* or biallelic variants associated with her phenotype were identified by ES.

### ARHGAP42 is highly expressed in the lung of human neonates and mice at postnatal (P) day 7–10

Publicly available RNA-seq data (LungMAP database, https://lungmap.net/) indicate that ARHGAP42 expression is high in the lung of human neonates and mice at P7-10 (S2A and S2B Fig). In addition, single-cell RNA-seq data from fetal lung tissues [8] (https://descartes.brotmanbaty.org/) show ARHGAP42 expression in stromal, vascular endothelial, bronchiolar and alveolar epithelial cells, myeloid, and lymphoid cells (S2C Fig).

### *ARHGAP42* stop-gain variant leads to exon 5 skipping

To evaluate the effect of *ARHGAP42* stop-gain variant, total RNA was extracted from Epstein-Barr virus-transformed lymphoblastoid cell lines (EBV-LCLs) of the proband, both parents, and an age-matched control. *ARHGAP42* cDNA sequences including the pre-variant, in-variant, and post-variant regions were amplified by PCR (primers shown in S2 Table). Agarose gel electrophoresis (AGE) results (Fig 3D) displayed a clear band in both the pre-variant and post-variant regions in the family and control, indicating that their transcription was not affected by the variant. However, all three family members exhibited two bands (196 and 298 bp) in the in-variant region, whereas the control showed only one (298 bp). The smaller 196 bp band was due to the skipping of exon 5 (ΔExon5), confirmed by Sanger sequencing. This band was much stronger in the proband compared to parents, suggesting a predominance of *ARHGAP42* ΔExon5 transcript. Quantitative real-time PCR (qRT-PCR) analysis of the *ARHGAP42* mRNA expression from EBV-LCLs of the proband, both parents, and an age-matched control confirmed the AGE findings (S3 Fig). A schematic of ARHGAP42 functional domains carrying this variant was shown in Fig 3E.

To verify the importance of *ARHGAP42* exon 5 in lung tissue, RNA was extracted from mouse lung tissues of different ages. The AGE of *ARHGAP42* RT-PCR products after cDNA synthesis from mouse lung tissue (10 days to eight months old) is shown in S4A Fig, and the *ARHGAP42* exon 5 was present in all the samples. In addition, the exon 5 was also present in the skeletal muscle tissues extracted from 10 days to one month old mice (S4B Fig). Sanger sequencing (S4C Fig) confirmed the exon 5 sequence of *ARHGAP42*, and demonstrated a high degree of sequence homology of exon 5 between human and mouse.

### *ARHGAP42* stop-gain variant leads to reduced ARHGAP42 expression

**Fig 4:**
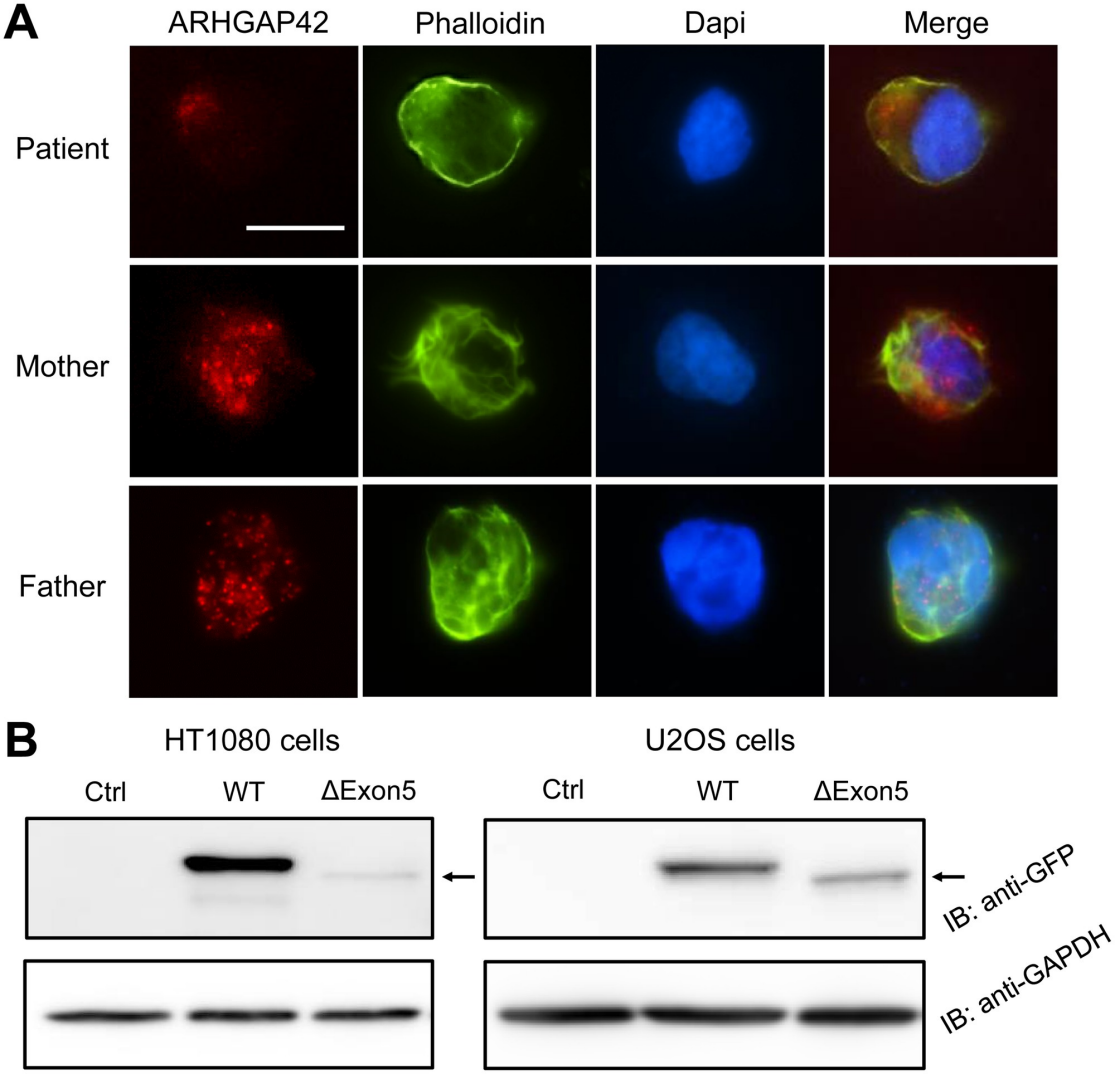
*ARHGAP42* stop-gain variant causes reduced ARHGAP42 expression. (A) Immunofluorescence of ARHGAP42 in the patient and her parents lymphoblastoid cells. Lymphoblastoid cells were fixed, permeabilized, and stained with DAPI (blue), phalloidin (green), and ARHGAP42 (red) (scale bar 10 μm); (B) Expression of control, GFP-ARHGAP42 WT and GFP-ARHGAP42 ΔExon5 in transfected HT1080 and U2OS cells by immunoblotting (black arrow indicates a lower level of ARHGAP42 ΔExon5 protein).

To assess the effect of *ARHGAP42* stop-gain variant on protein expression, we performed immunostaining against ARHGAP42 in the patient EBV-LCLs. Immunofluorescence analyses demonstrated a lower expression of ARHGAP42 in the patient than that in her parents (Fig 4A). Immunoblotting was not applicable due to a lack of good-quality antibodies.

We have shown that this stop-gain variant leads to exon 5 skipping in the patient. To evaluate the effects of *ARHGAP42* ΔExon5 at the protein level, immunoblotting in HT1080 and U2OS cells transfected with *ARHGAP42* ΔExon5 construct were undertaken. Both U2OS and HT1080 cells were transfected with GFP-*ARHGAP42* WT or GFP-*ARHGAP42* ΔExon5 and successfully transfected cells were selected by fluorescence-activated cell sorter (S5 Fig). Both cell lines showed less ARHGAP42-ΔExon5 expression than ARHGAP42-WT (Fig 4B).

### *ARHGAP42* stop-gain variant contributes to enhanced RhoA activity

**Fig 5:**
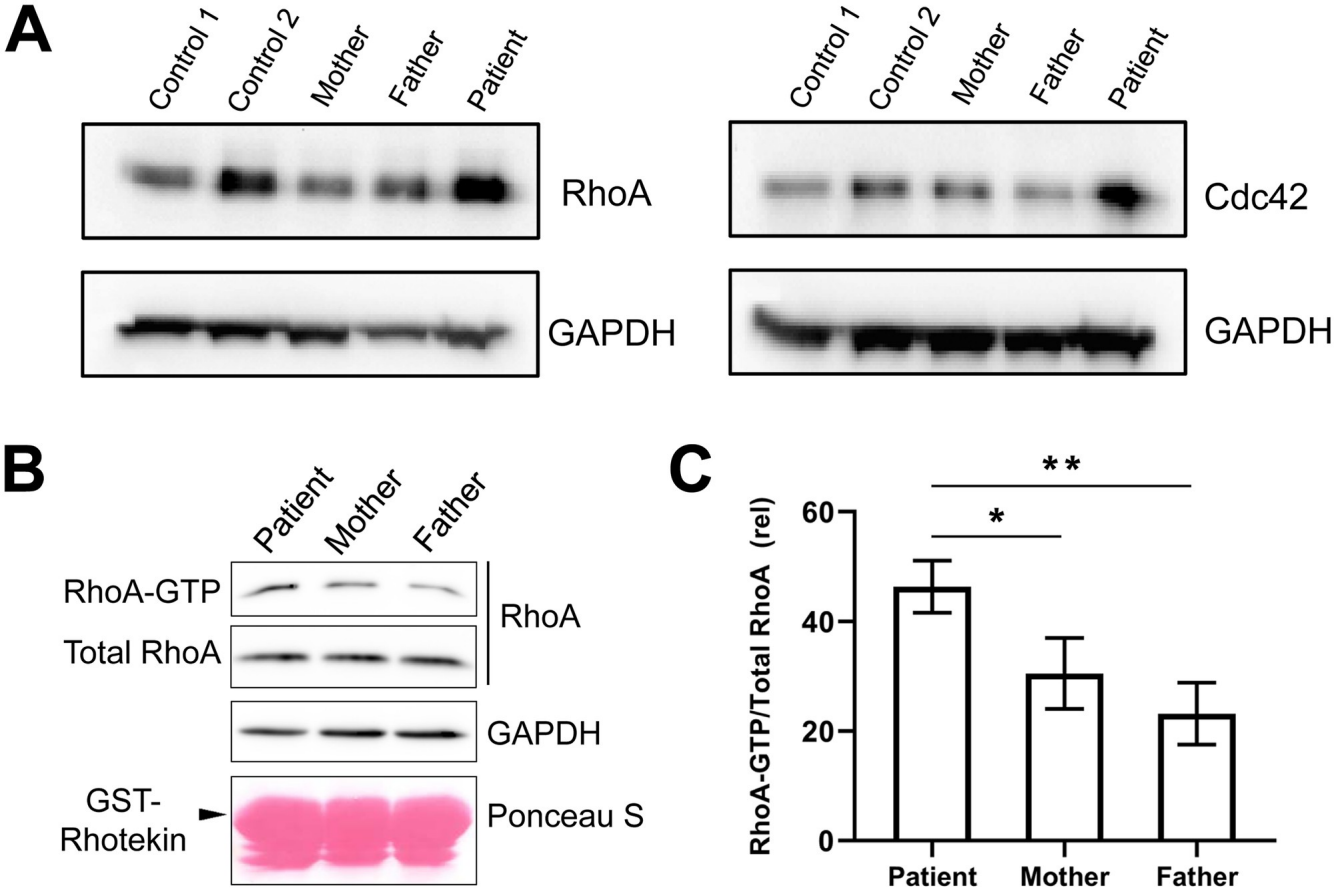
*ARHGAP42* stop-gain variant contributes to enhanced RhoA activity and Cdc42 expression. (A) Expression of RhoA and Cdc42 in the patient’s EBV-LCLs compared with her parents and healthy controls by immunoblotting; (B) Measurement of RhoA activity by GST-Rhotekin-RBD (Rho binding domain) pull-down assay in the patient and her parents. The level of RhoA-GTP was determined by western blot with anti-RhoA antibody following a pull-down assay; (C) The RhoA-GTP data are expressed as the mean ± SD of three independent experiments. *p<0.05; **p<0.01.

ARHGAP42 is a member of the RhoGAP family, and it acts as a GAP for RhoA and Cdc42. First, we evaluated the levels of RhoA and Cdc42 protein in the patient’s EBV-LCLs. Both were higher in the patient than that in the parents and healthy controls by immunoblotting (Fig 5A). To further test the effects of *ARHGAP42* stop-gain variant on the RhoA activity, a GST-Rhotekin-RBD (Rho binding domain) pull-down assay was performed on the patient’s EBV-LCLs (Fig 5B). The RhoA-GTP levels were significantly increased in the patient relative to both parents (Fig 5C).

### BAR function is preserved in *ARHGAP42* ΔExon5

ARHGAP42 has four major domains, including the BAR (Bin/amphiphysin/Rvs), GAP, PH (pleckstrin homology) and SH3 (SRC Homology 3) domain. Exon 5 encodes for part of the BAR domain, a highly conserved protein dimerization domain, which acts as membrane curvature sensor by preferentially binding to curved membranes [9,10]. We evaluated BAR activity in U2OS cells expressing GFP-ARHGAP42 ΔExon5, GFP-ARHGAP42 ΔBAR, GFP-ARHGAP42 ΔGAP and GFP-ARHGAP42 WT for membrane tubulation study. The membrane tubulations were significantly inhibited in the ARHGAP42 ΔBAR cells, but markedly increased in the ARHGAP42 ΔGAP cells. However, we did not observe significant difference between ARHGAP42 ΔExon5 and ARHGAP42 WT cells, indicating that the BAR domain function was preserved in the patient (S6 Fig).

## Discussion

In this study, we identified a patient with chILD, hypertension, and immune abnormalities caused by a rare homozygous stop-gain variant (p.(Glu157Ter)) in exon 5 of *ARHGAP42*. This variant leads to skipping of exon 5 but no evidence of nonsense mediated mRNA decay was found on RNA studies of the patient’s EBV-LCLs. To further evaluate the effects of exon 5 skipping on the protein expression, we transfected the U2OS and HT1080 cells with GFP-ARHGAP42 ΔExon5 construct, and found that ARHGAP42 levels were markedly reduced compared to WT controls. This was consistent with reduced expression of ARHGAP42 in the patient’s EBV-LCLs by immunofluorescence. Further, we found the RhoA level and activity in the patient’s EBV-LCLs to be significantly increased compared to controls, as an evidence of reduced ARHGAP42 function. These results suggest that the homozygous stop-gain variant in the patient leads to a decreased level of ARHGAP42 protein and its loss of function.

In addition, we tested the effect of exon 5 deletion on the BAR domain function. The BAR domain of ARHGAP42 is autoinhibitory toward GAP, and its deletion is associated with a reduction of RhoA activity due to increased GAP function [11]. However, when we expressed ARHGAP42 ΔExon5 in the U2OS cells, the membrane tubulation activity was preserved, indicating an intact BAR domain function.

### ARHGAP42 haploinsufficiency leads to systemic hypertension in human patients

A hypomorphic *Arhgap42*-KO mouse model has been described that exhibited significant hypertension but no other overt phenotypes observed for up to 6 months [1]. A recent study also found that haploinsufficiency of ARHGAP42 leads to an age-dependent hypertension in humans [12]. As mentioned, this patient has systemic hypertension along with both parents and paternal grandfather, all heterozygous for the identified variant. In addition, on most recent echocardiogram, the proband has borderline increase in left ventricular diastolic posterior wall thickness with a z-score of 2.1, which may be secondary to the systemic hypertension.

Previous studies of ARHGAP42 in vascular SMCs have demonstrated that ARHGAP42 controls BP by inhibiting RhoA-dependent contractility [6,13]. ARHGAP42 depletion is associated with significantly increased RhoA activity, which may lead to Rho-kinase (ROCK)-dependent inhibition of myosin phosphatase, an enzyme that dephosphorylates MLC. Indeed, both aortic segments and peripheral resistance vessels from ARHGAP42-deficient mice exhibited elevated levels of phosphorylated MLC relative to control mice [1]. Treatment with ROCK inhibitor completely abrogated the agonist-induced increases in systolic BP in ARHGAP42-deficient mice [1]. In addition to the RhoA/ROCK-mediated contractility, RhoA has a major impact on the transcription of numerous contractile genes [6]. Moreover, RhoA/ROCK activation has been reported to be associated with adult-type fibrotic lung diseases [14–17]. RhoA activation promotes pulmonary fibrotic processes by activating pMLC to enhance stress fiber and focal adhesion formation, and also cell contraction, thus affecting actin filament assembly and actomyosin contraction among epithelial cells, fibroblasts, endothelial cells, and macrophages [18].

### A potential link between ARHGAP42 deficiency and the development of chILD

The histologic phenotype of the patient’s lung is of note. Small airways contained prominent smooth muscle and obstructive-like features, with accumulation of intra-luminal and intra-alveolar macrophages. Architectural remodeling included extension of airway smooth muscle into alveolar septa as well as enlargement/maldevelopment of subpleural airspaces. The concentric medial hypertrophy of pulmonary arteries identified is a feature of pulmonary hypertension. Patchy pulmonary interstitial glycogenosis, observed herein, is abnormal differentiation of the pulmonary mesenchyme, often associated with developmental lung abnormalities and pulmonary arterial hypertension (PAH). Further, maldevelopment with deficient alveolarization leading to a decreased size of the pulmonary capillary bed may also contribute to pulmonary arterial remodeling in this patient.

In our patient, evidence of prominent mural smooth muscles in small airways and alveolar septa, and concentric hypertrophy in pulmonary arteries along with an increase in RhoA/ROCK signaling in her EBV-LCLs suggests a mechanistic link between ARHGAP42 deficiency and the development of chILD syndrome. We postulate that ARHGAP42 deficiency enhances downstream RhoA/ROCK signaling and leads to increased SMC contractility in the airway and pulmonary arterial SMCs, which may explain the chILD phenotype.

The histopathological findings of concentric hypertrophy in pulmonary arteries can be associated with the development of PAH. While the serial echocardiograms have shown no evidence of pulmonary hypertension, the proband will continue to be evaluated for this condition. Further, both parents and the paternal grandfather (all carriers for this stop-gain variant) have history of exercise-induced asthma, which may be linked to the smooth muscle hypertrophy seen in the proband. In our patient, it is difficult to exclude chronic airway inflammation, possibly related to immune dysregulation, as a factor contributing to the airway smooth muscle remodeling, but this is less likely given the presentation in early infancy and overall improvement with growth, as well as the absence of cellular inflammatory airway infiltrates on lung biopsy. Evaluating the small airways of the *Arhgap42*-KO mouse model may provide further evidence linking ARHGAP42 with asthma or other chronic airway disease.

### ARHGAP42 deficiency may be associated with impaired immune responses through altered RhoA signaling and actin dynamics

The patient’s immune findings were notable for chronic lymphocytosis with intermittent neutrophilia. Elevation in the total number of CD4^+^ and CD8^+^ T cells, B cell and NK cells was roughly proportional, suggesting that multiple cell types were equally affected. While evaluation of the patient’s humoral immunity was limited, the absence of some specific antibody responses and decreased circulating plasmablasts and transitional B cells (populations associated with generation of high-affinity antibody) may also suggest impairment in generation of antigen-specific IgG. The patient’s clinical history of persistent cutaneous warts, could also suggest inability to clear HPV infection.

Impaired immune responses have been observed in primary immunodeficiencies associated with disrupted regulation of actin dynamics, such as Wiskott-Aldrich Syndrome (WASP), Coronin1A, Rac2, and ARHGEF1. The rapid assembly and disassembly of actin filaments is required for cell motility, adhesion, cell-to-cell interactions, and many other critical functions of immune cells. Patients may exhibit cellular, humoral or innate immune defects, or a combination, depending upon the cell types affected. While immune deficiency associated with defects in ARHGAP42 has not previously been reported, ARHGAP42 and RhoGTPases are important regulators of actin cytoskeletal dynamics [19,20]. Disrupted regulation of cytoskeletal rearrangements can have significant consequences upon cellular motility.

Previous reports have demonstrated that loss of the ARHGAP42 BAR inhibitory domain is associated with decreased RhoA activity and increased cellular motility *in vitro* [11], and increased expression of ARHGAP42 in cancer cells is associated with increased cell migration and invasion [21,22]. As the *ARHGAP42* stop-gain variants produces the opposite effect (increased RhoA activity), we hypothesize that the overall effect upon leukocyte migration may be deleterious. This may explain the consistent observation of lymphocytosis in the proband, as the large numbers of lymphocytes with the *ARHGAP42* variant may be circulating in the blood, due to an impaired ability to exit the blood vessels (as observed in CD18 deficiency (Leukocyte adhesion deficiency type-1 (LAD-1)). Clearance of cutaneous HPV infection may also be affected by disrupted actin dynamics. Clearance of warts requires the presence of appropriately activated immune effectors at the site, which may be reduced in the proband due to decreased capacity of the effector cells to efficiently traffic to the location.

In summary, we have identified a homozygous stop-gain variant in *ARHGAP42* in a child with chILD, hypertension, and immune abnormalities. Although haploinsufficiency of ARHGAP42 has been previously linked to hypertension at adulthood [12], this is the first time that a homozygous stop-gain variant in *ARHGAP42* has been linked to chILD and immune problems. Our findings suggest that ARHGAP42 loss-of-function causes increased RhoA activity, which may lead to increased SMC contractility in the lung and disrupted regulation of actin dynamics in the immune cells. The expression pattern of ARHGAP42 in the lung, highest in early postnatal life and decreasing with age, is consistent with the course of disease in our patient. This study is the first to document ARHGAP42 deficiency-associated lung disease and to implicate the RhoA/ROCK pathway as the potential underlying mechanism.

## Methods

### Ethics statement

Written consent was obtained from the parents in accordance with the ethical standards of the participating institutional review board (IRB) at Boston Children’s Hospital. Clinical records were reviewed. The IRB at Boston Children’s Hospital approved the human cell work under the protocol 10-02-0253.

### Pathologic examination

Light microscopic examination of the patient’s lung biopsy tissue, processed routinely for clinical evaluation, was conducted. Stains examined included hematoxylin and eosin (H&E), Masson trichrome, Miller elastic, periodic acid-Schiff with diastase, and reticulin; positive controls were run in parallel.

### Exome sequencing

The proband, both parents, and extended family were enrolled in the Gene Discovery Core of the Manton Center for Orphan Disease Research, an IRB-approved research protocol (10-02-0053) at Boston Children’s Hospital. The QIAmp DNA Mini Kit (Qiagen, MD, USA) was used for genomic DNA extraction from peripheral blood lymphocytes. Exome sequencing data was generated, and Fastq data filtered and aligned, and variants filtered and annotated by the Codified Genomics and the Variant Explorer (VExP) pipelines as described previously [23,24].

### Cell culture of Epstein-Barr virus-transformed lymphoblastoid cell lines (EBV-LCLs)

Human B-lymphocytes were immortalized by transformation with the EBV according to established procedures [25]. EBV-LCLs of the patient and the parents were cultured in RPMI-1640 medium (ThermoFisher) supplemented with 15% fetal bovine serum (FBS, ThermoFisher), and 1% penicillin and streptomycin (ThermoFisher) at 37 °C in a 5% CO_2_ atmosphere. Cells were maintained at a concentration between 4 x 10^5^–1 x 10^6^ cells/mL and expanded as needed.

### RNA analyses

RNA was extracted from the LCLs of the proband and both parents, and complementary DNA (cDNA) was synthesized using the Superscript III kit (ThermoFisher, MA, USA) according to the manufacturer’s protocol. The cDNA was amplified by polymerase chain reaction (PCR) using three sets of primers (S2 Table) and analyzed by agarose gel electrophoresis. Quantitative real-time PCR was performed using the SYBR Green qPCR kit (ThermoFisher). Specific primers were designed near variant regions for *ARHGAP42* and for *GUSB* as the reference gene for normalization (S3 Table). Reactions were run on the Quant Studio 3 (ThermoFisher) in triplicates. The results presented are the mean of three independent experiments. The relative expression of *ARHGAP42* was normalized to *GUSB* and calculated using the comparative cycle threshold (2^-ΔΔCt^) method. Statistical significance was assessed by a standard unpaired, two-tailed Student’s t-test. *P*-values under 0.05 was considered significant.

### Immunofluorescence

EBV-LCL cells were centrifuged at 320 × g for 5 min. Supernatant was discarded and the cells were resuspended in phosphate-buffered saline (PBS). Cells were allowed to settle and adhere for 5 min in 1 mg/ml poly-L-lysine coated coverslips (Sigma-Aldrich, MO, USA). Twenty-five percent of the cell suspension volume of 4% paraformaldehyde (PFA) was then added for a 2-min incubation at room temperature (RT). The solution was removed and replaced with fresh 4% PFA for 30 min at RT without agitation. Cells were washed gently using PBS three times before being subjected to standard immunofluorescent staining with anti-ARHGAP42 antibody (1:500 dilution, from Dr. Joan M. Taylor’s lab, UNC, USA), vinculin antibody (1:500 dilution, MilliporeSigma, MA, USA), anti-Rabbit IgG (H+L) secondary antibody-Alexa Fluor 594 (1:2000 dilution, ThermoFisher) or Alexa Fluor 488 Phalloidin (1:400 dilution, ThermoFisher). Cells were coverslipped using Vectashield Mounting Medium with DAPI (Vector Laboratories, CA, USA). Staining was evaluated using a Nikon Eclipse 90i microscope using NIS-Elements AR software (Nikon Instruments Inc., NY, USA).

### *ARHGAP42* cDNA cloning and mutagenesis

Human *ARHGAP42* cDNA with C-terminal Flag tag was purchased from GenScript in pcDNA3.1 +/C-(K)-DYK. Mutagenesis of *ARHGAP42* wild type (WT) to ΔExon5, ΔBAR and ΔGAP was performed using the WHOPS (whole plasmid synthesis) approach with the primers listed in S4 Table. Briefly, PCR with the respective primers, Phusion DNA polymerase (New England Biolabs) and *ARHGAP42* WT as a template was run for 18 cycles at 95°C for 50 s, 53°C for 50 s and 72°C for 6 min. After PCR, 5 U of DpnI (New England Biolabs) were added to the reaction, incubated 2 h in 37°C, transformed into TOP10 bacteria and grown on a plate overnight in 37°C. The bacteria transformed with *ARHGAP42* WT construct had to be grown on a plate for two days in 30°C. Subsequently, colony PCR was performed to identify the clones carrying the correct full-length version of *ARHGAP42* WT encoding plasmid. Positive clones were then grown for 2–3 days in 30°C.

### Transfection of HT1080 and U2OS cells

For stable transfection of HT1080 cells, plasmids with individual variants of GFP-*ARHGAP42* were linearized and transfected into HT1080 cells using PEI (Polysciences). 48 h after transfection, the cells were selected for one week with G418 and FACS sorted. U2OS cells were transiently transfected using PEI (Polysciences).

### Immunoblotting

Lysates from LCLs were prepared with a modified RIPA buffer containing protease and phosphatase inhibitors. Protein concentration was determined with use of a colorimetric BCA assay. Proteins were transferred to PVDF membrane and blocked for an hour at room temperature in 5% Blotting-Grade blocker reagent in PBS plus 0.2% Tween-20 (PBST). The membrane was rinsed briefly with PBST, and blotted with Rho A (Cell Signaling Technology, cat# 2117S), Cdc42 (Cell Signaling Technology, cat# 2466S), and anti-mouse antibody against GAPDH (Abcam, cat#9485) overnight at 4°C on a rocker. Blots were washed in PBST, followed by incubation with Goat HRP-anti-Rabbit antibody. Blots were visualized with Super Signal West Pico PLUS Chemiluminescent Substrate in a Gel Doc imaging System using Quantity One software.

### RhoA-GTP pull-down assay

The lysates were cleared by centrifugation (13 000×g, 13 min) and equalized for the total protein amount (DC Protein Assay, BioRad). Agarose-bound GST Rhotekin (20 μg) was added into each lysate and rotated in 4°C for 40 min. After incubation, the beads were washed three times in lysis buffer and resuspended in SDS-PAGE sample buffer. The samples were resolved using SDS-PAGE and analyzed by immunoblotting with respective antibodies: RhoA (67B9) mAb (2117, Cell Signaling Technology) and GAPDH Loading Control Monoclonal Antibody (GA1R, MA5-15738, Invitrogen).

### Analysis of membrane tubulations

U2OS cells were seeded on fibronectin-coated coverslips and transfected with the individual variants of GFP-*ARHGAP42* using PEI (Polysciences). After 48 h the cells were fixed with 4% PFA and actin was stained using Alexa Fluor 594 Phalloidin (1:200 dilution, ThermoFisher). At least 100 cells per variant were scored in three independent experiments for the presence of membrane tubulations using Nikon Eclipse TE 2000-S with NIS-Elements AR software (Nikon Instruments Inc., NY, USA).

### Statistical analysis

*P*-values indicate statistical significance determined by one-way ANOVA followed by Tukey’s multiple comparisons test.

## Supporting information

S1 Table Ambulatory blood pressure levels of the proband fell within the hypertensive range. The mean 24-hour systolic blood pressure was at the 95th percentile and the mean 24-hour diastolic ambulatory blood pressure fell above the 95th percentile for sex-age. BP load was elevated (25–50% elevated) for systolic blood pressures. BP load was significantly elevated (>50% elevated) for diastolic blood pressures. Diurnal variation (nocturnal “dipping”) for systolic and diastolic blood pressures was normal.
(XLSX)

S2 Table Primers sequences used for PCR amplification of human and mouse *ARHGAP42* exons near variant region. (XLSX)

S3 Table Primers sequences used for quantitative real-time PCR amplification of human *ARHGAP42* exons near variant region, and *GUSB* used as the reference gene for normalization. (XLSX)

S4 Table Primers sequences used for mutagenesis of *ARHGAP42* WT to ΔExon5, ΔBAR and ΔGAP by whole plasmid synthesis approach. (XLSX)

S1 Fig Immune abnormalities in the proband. Immune cell counts including total WBC (left), lymphocytes (middle) and neutrophils (right) are shown by age in years, with the maximum normal for age (shaded). The proband had an initial leukocytosis at birth, which briefly resolved but then has persisted after 2 years of age. Lymphocytosis (middle) was more consistent. See S1 Data.
(TIF)

S2 Fig ARHGAP42 is highly expressed in the lung of human neonates and mice at postnatal (P) day 7–10. RNA-seq data from the LungMAP database showing ARHGAP42 expression patterns in (A) human and (B) mouse lung by time. E: embryonic day; P: postnatal day. (C) Single-cell RNA-seq data from human fetal lung cells showing ARHGAP42 expression in stromal, vascular endothelial, bronchiolar and alveolar epithelial, myeloid, and lymphoid cells. See S2 Data.
(TIF)

S3 Fig Quantitative real-time PCR analysis of the *ARHGAP42* mRNA expression in the Epstein-Barr virus-transformed lymphoblastoid cell lines of the patient, parents and control using different primer pairs specific for exon 5 and after exon 5 regions. **p<0.01. See S3 Data.
(TIF)

S4 Fig *ARHGAP42* exon 5 is preserved in mouse lung and skeletal muscle tissues at different ages. (A) Agarose gel electrophoresis of *ARHGAP42* RT-PCR products after RNA extraction and cDNA synthesis in mouse lung tissue at different ages (10 days, one month, three months and eight months) and (B) mouse skeletal muscle tissues from 10 days to one month; (C) Sanger sequence confirms the present of *ARHGAP42* exon 5 from (A). m1: 10 days; m2: 10 days; m3: 1 month; m4: 1 month; m5: 3 months; m6: 8 months. See S4 Data.
(TIF)

S5 Fig GFP-*ARHGAP42* WT or GFP-*ARHGAP42* ΔExon5 stable transfection in U2OS and HT1080 cells and selected by FACS for immunoblotting experiments. FACS, fluorescence-activated cell sorter.
(TIF)

S6 Fig BAR function is preserved in U2OS cells expressing GFP-*ARHGAP42* ΔExon5 construct. Representative U2OS cells expressing GFP-*ARHGAP42* WT, GFP-*ARHGAP42* ΔExon5, GFP-*ARHGAP42* ΔBAR and GFP-*ARHGAP42* ΔGAP constructs for membrane tubulation study. The cells were fixed and visualized for GFP fluorescence (left panel, scale bar 10 μm). Quantitative analysis of membrane tubulation induced by *ARHGAP42* variants (right panel). Values are mean ± SD from three independent transfection experiments, with at least 100 cells scored per variant. Statistical significance was determined by one-way ANOVA followed by Tukey’s multiple comparisons test. ****p<0.001, ns: not statistically significant. See S5 Data.
(TIF)

S1 Data Immune cell counts including total WBC, lymphocytes and neutrophils are shown by age in years. (XLSX)

S2 Data **S2A**. RNA-seq data from the LungMAP database showing ARHGAP42 expression patterns in human lung by time. **S2B**. RNA-seq data from the LungMAP database showing ARHGAP42 expression patterns in mouse lung by time. E: embryonic day; P: postnatal day.
(XLSX)

S3 Data Quantitative real-time PCR analysis of the *ARHGAP42* mRNA expression in the Epstein-Barr virus-transformed lymphoblastoid cell lines of the patient, parents and control using different primer pairs specific for exon 5 and after exon 5 regions. (XLSX)

S4 Data Sanger sequencing alignment file of *ARHGAP42* exon 5 in mice at different ages. (ZIP)

S5 Data Quantitative analysis of membrane tubulation induced by *ARHGAP42* variants. (XLSX)
