# Supplementary figures and images for: A homozygous stop-gain variant in ARHGAP42 is associated with childhood interstitial lung disease, systemic hypertension, and immunological findings
Source: PLoS Genet. 2021 Jul 7;17(7):e1009639. doi: 10.1371/journal.pgen.1009639 (PMC8289122; doi:10.1371/journal.pgen.1009639)

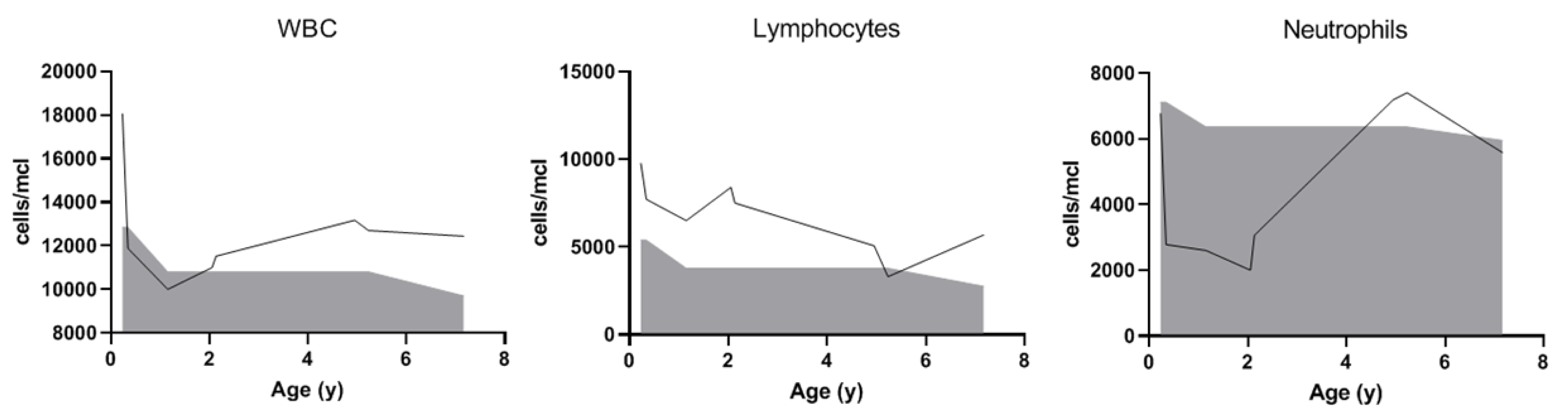

Supplement: S1 Fig — Immune cell counts including total WBC (left), lymphocytes (middle) and neutrophils (right) are shown by age in years, with the maximum normal for age (shaded). The proband had an initial leukocytosis at birth, which briefly resolved but then has persisted after 2 years of age. Lymphocytosis (middle) was more consistent. See S1 Data. (TIF) [file pgen.1009639.s005.tif]

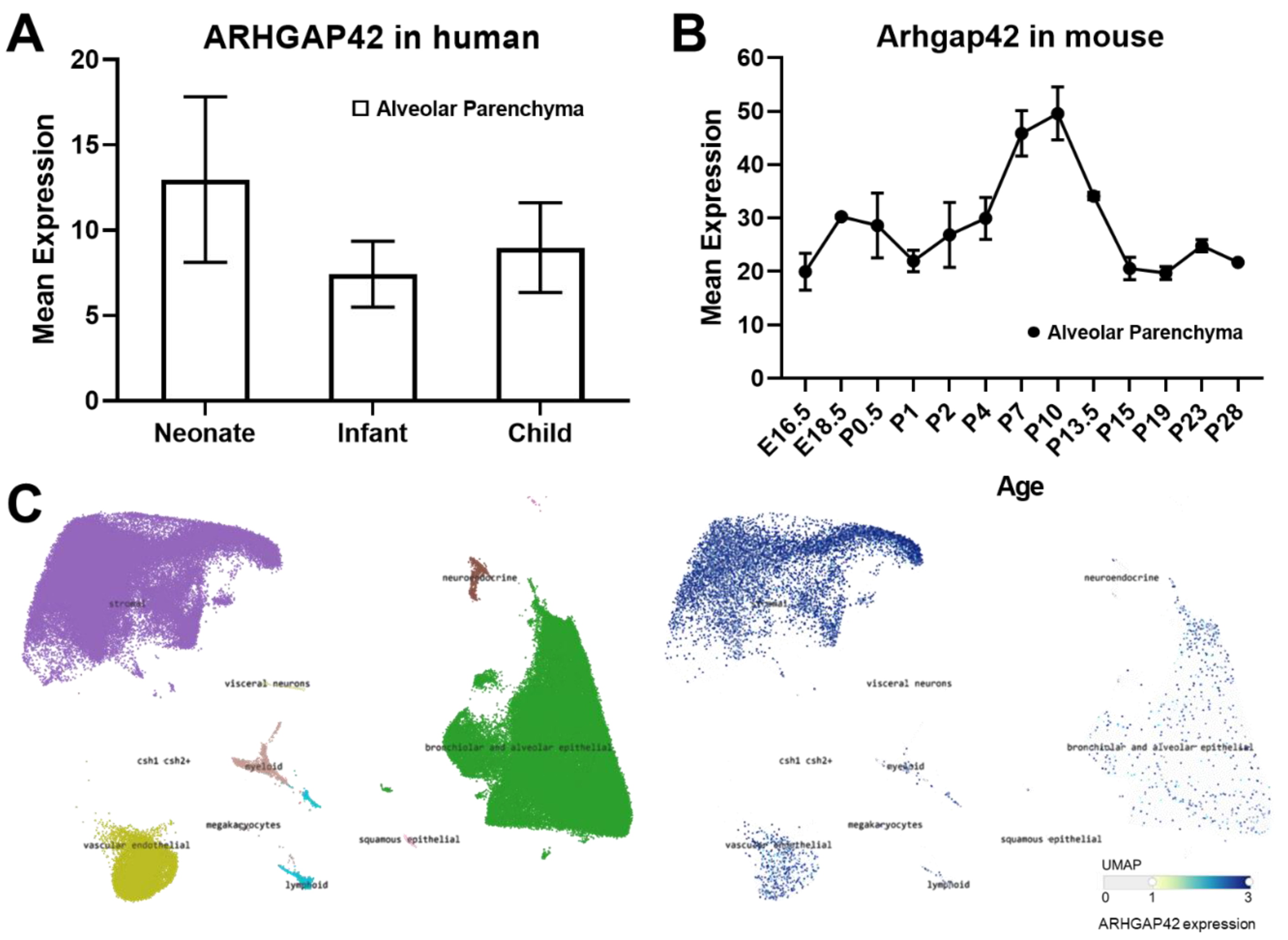

Supplement: S2 Fig — RNA-seq data from the LungMAP database showing ARHGAP42 expression patterns in (A) human and (B) mouse lung by time. E: embryonic day; P: postnatal day. (C) Single-cell RNA-seq data from human fetal lung cells showing ARHGAP42 expression in stromal, vascular endothelial, bronchiolar and alveolar epithelial, myeloid, and lymphoid cells. See S2 Data. (TIF) [file pgen.1009639.s006.tif]

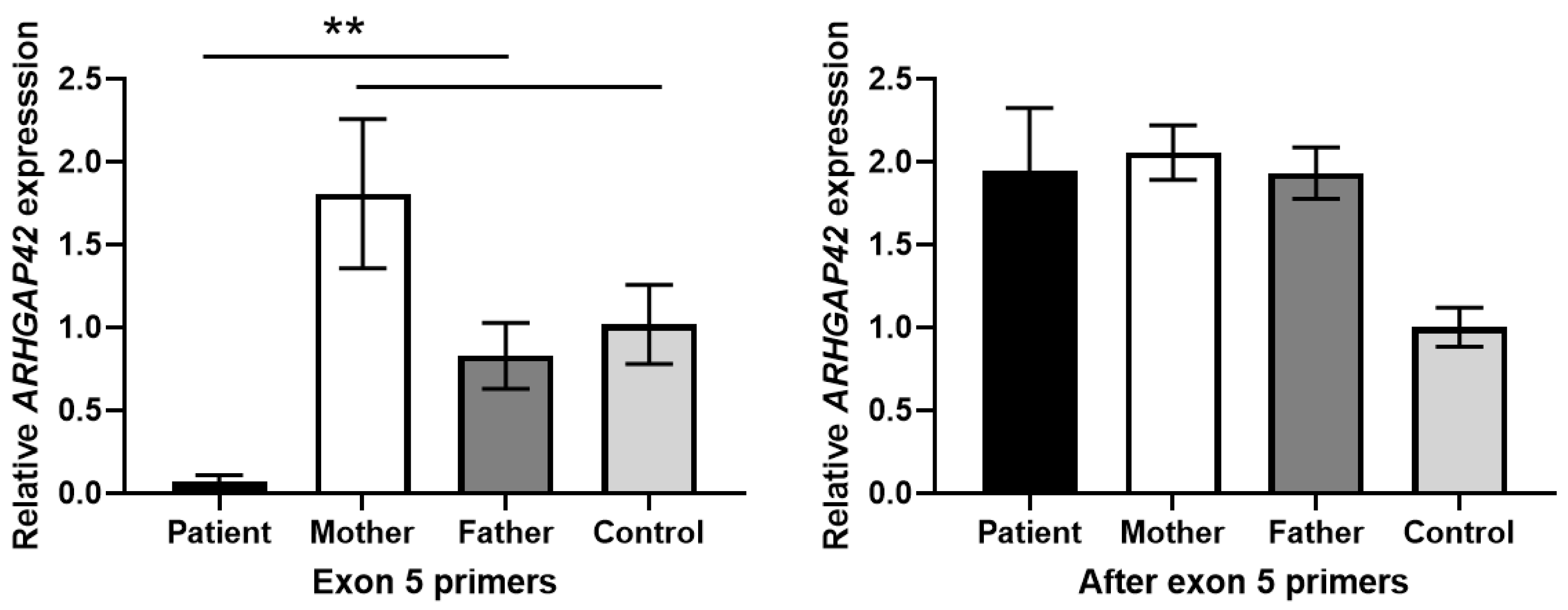

Supplement: S3 Fig — **p<0.01. See S3 Data. (TIF) [file pgen.1009639.s007.tif]

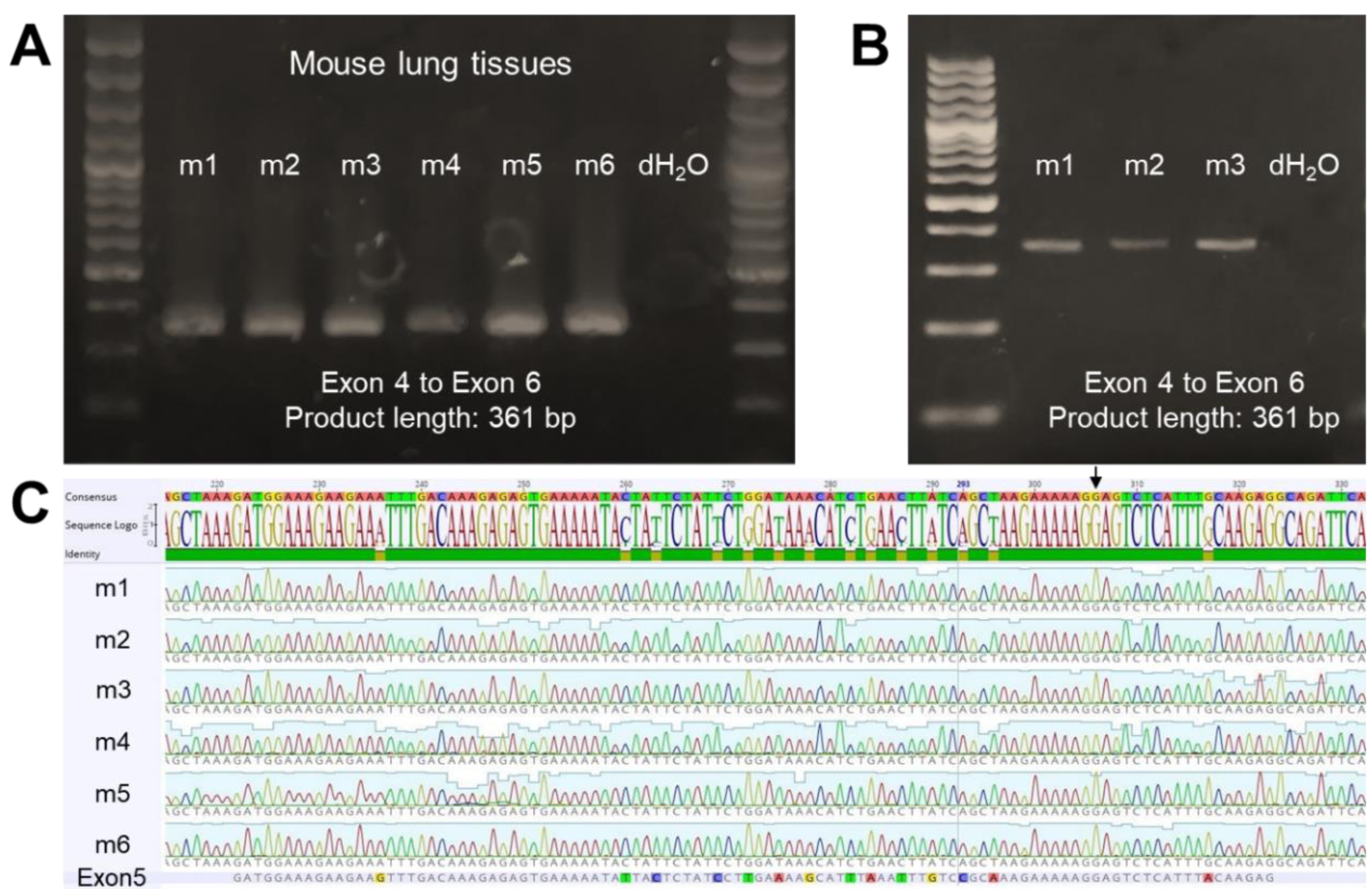

Supplement: S4 Fig — (A) Agarose gel electrophoresis of ARHGAP42 RT-PCR products after RNA extraction and cDNA synthesis in mouse lung tissue at different ages (10 days, one month, three months and eight months) and (B) mouse skeletal muscle tissues from 10 days to one month; (C) Sanger sequence confirms the present of ARHGAP42 exon 5 from (A). m1: 10 days; m2: 10 days; m3: 1 month; m4: 1 month; m5: 3 months; m6: 8 months. See S4 Data. (TIF) [file pgen.1009639.s008.tif]

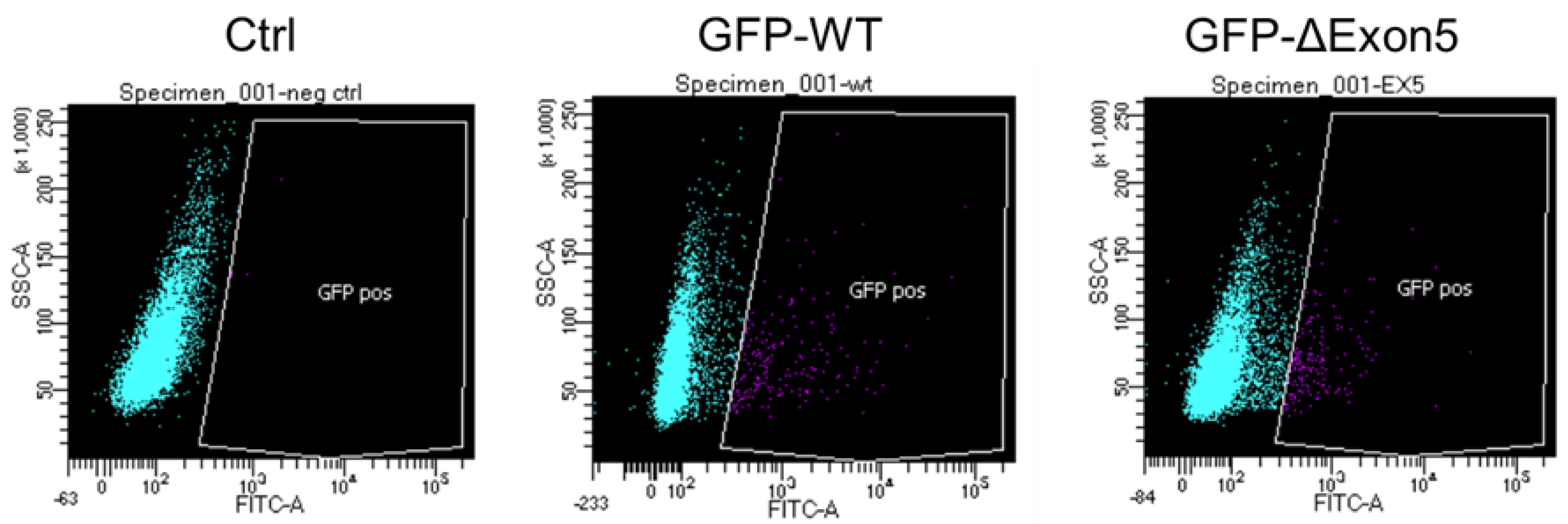

Supplement: S5 Fig — FACS, fluorescence-activated cell sorter. (TIF) [file pgen.1009639.s009.tif]

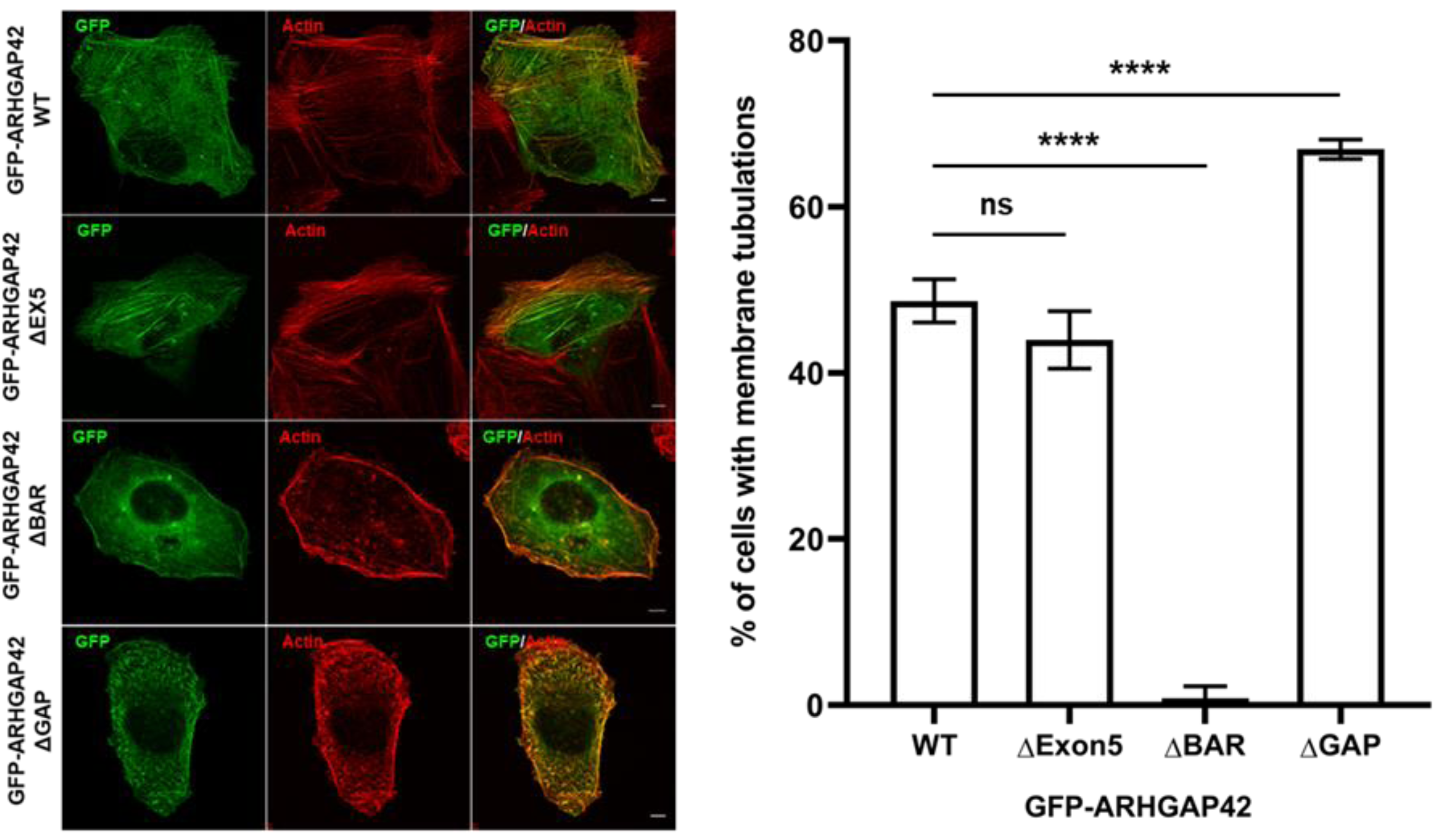

Supplement: S6 Fig — Representative U2OS cells expressing GFP-ARHGAP42 WT, GFP-ARHGAP42 ΔExon5, GFP-ARHGAP42 ΔBAR and GFP-ARHGAP42 ΔGAP constructs for membrane tubulation study. The cells were fixed and visualized for GFP fluorescence (left panel, scale bar 10 μm). Quantitative analysis of membrane tubulation induced by ARHGAP42 variants (right panel). Values are mean ± SD from three independent transfection experiments, with at least 100 cells scored per variant. Statistical significance was determined by one-way ANOVA followed by Tukey’s multiple comparisons test. ****p<0.001, ns: not statistically significant. See S5 Data. (TIF) [file pgen.1009639.s010.tif]
